# A Role of Microtubules in Oligodendrocyte Differentiation

**DOI:** 10.3390/ijms21031062

**Published:** 2020-02-05

**Authors:** Bo Yoon Lee, Eun-Mi Hur

**Affiliations:** 1 Division of Bio-Medical Science & Technology, KIST School, Korea University of Science and Technology (UST), Seoul 02792, Korea; byleeshine@gmail.com; 2 Department of Neuroscience, College of Veterinary Medicine, Research Institute for Veterinary Science, and BK21 PLUS Program for Creative Veterinary Science Research, Seoul National University, Seoul 08826, Korea

**Keywords:** mouse oligodendrocyte, survival, differentiation, microtubule

## Abstract

Oligodendrocytes are specialized cells that myelinate axons in the central nervous system. Defects in oligodendrocyte function and failure to form or maintain myelin sheaths can cause a number of neurological disorders. Oligodendrocytes are differentiated from oligodendrocyte progenitor cells (OPCs), which extend several processes that contact, elaborate, and eventually wrap axonal segments to form multilayered myelin sheaths. These processes require extensive changes in the cytoarchitecture and must be regulated by reorganization of the cytoskeleton. Here, we established a simple protocol to isolate and differentiate mouse OPCs, and by using this method, we investigated a role of microtubules (MTs) in oligodendrocyte differentiation. Oligodendrocytes developed a complex network of MTs during differentiation, and treatment of differentiating oligodendrocytes with nanomolar concentrations of MT-targeting agents (MTAs) markedly affected oligodendrocyte survival and differentiation. We found that acute exposure to vincristine and nocodazole at early stages of oligodendrocyte differentiation markedly increased MT arborization and enhanced differentiation, whereas taxol and epothilone B treatment produced opposing outcomes. Furthermore, treatment of myelinating co-cultures of oligodendrocytes and neurons with nanomolar concentrations of MTAs at late stages of oligodendrocyte differentiation induced dysmyelination. Together, these results suggest that MTs play an important role in the survival, differentiation, and myelination of oligodendrocytes.

## 1. Introduction

Oligodendrocytes, the myelinating glia in the central nervous system (CNS), enable rapid conduction of electrical impulses and play an essential role in maintaining the long-term integrity of the underlying axons. Indeed, white matter deficits are associated with a wide range of neurodevelopmental disorders and neurodegenerative diseases [1,2,3,4]. Myelinating oligodendrocytes are generated from oligodendrocyte precursor cells (OPCs), which undergo remarkable morphological changes as they differentiate. OPCs send out numerous processes, which contact and wrap multiple axonal segments to form the tightly packed layers of the myelin sheaths [5,6]. 

In eukaryotic cells, cytoskeletal components are responsible for the formation, maintenance, and remodeling of cell shape. Transcriptomic analysis of developing oligodendrocytes has revealed that aside from genes encoding myelin proteins, one of the most heavily regulated genes during OPC development are those related to cytoskeleton remodeling [7]. Among the three main cytoskeleton, F-actin, intermediate filaments, and microtubules (MTs), oligodendrocytes are known to contain two, F-actin and MTs [8,9]. Previous studies have suggested that F-actin assembly mediates the initial protrusion of the motile leading edge during oligodendrocyte differentiation and that subsequent F-actin disassembly facilitates myelin sheet formation at later stages [5,6,10]. Compared to actin, little is known about the role of MTs in oligodendrocyte differentiation. A recent study has shown that tubulin polymerization promoting protein (TPPP) nucleates MTs in oligodendrocytes and that *Tppp* knockout mice exhibit hypomyelination and motor coordination defects [11], but how MTs contribute to oligodendrocyte differentiation and myelination remains largely enigmatic. 

The cytoskeletal structure of myelinating oligodendrocytes in vivo cannot be easily resolved under light microscopy because of the small dimensions and tight packing of myelin. To overcome this inherent difficulty, several methods have been developed to culture oligodendrocytes, and primary cultures of oligodendrocytes are now widely used to investigate the cell biological mechanism of oligodendrocyte differentiation. In culture, oligodendrocytes form flat sheets akin to an unrolled myelin sheath, and the molecular composition is quite similar to compact myelin in vivo [12]. Moreover, the timing of major myelin protein expression in vitro closely resembles that of in vivo [12,13]. Indeed, studies in culture have led to important findings regarding a role of F-actin assembly and disassembly during myelination [5,6]. Here, we have modified an existing method [14] to establish a simple protocol for isolating and differentiating mouse OPCs. By using this method, we investigated a role of MTs in oligodendrocyte differentiation. Our results suggest that oligodendrocytes develop a complex arborization of MTs during differentiation and that MT dynamics plays an essential role in oligodendrocyte differentiation and myelination.

## 2. Results

### 2.1. Purification and Differentiation of Mouse OPCs

**Figure 1:**
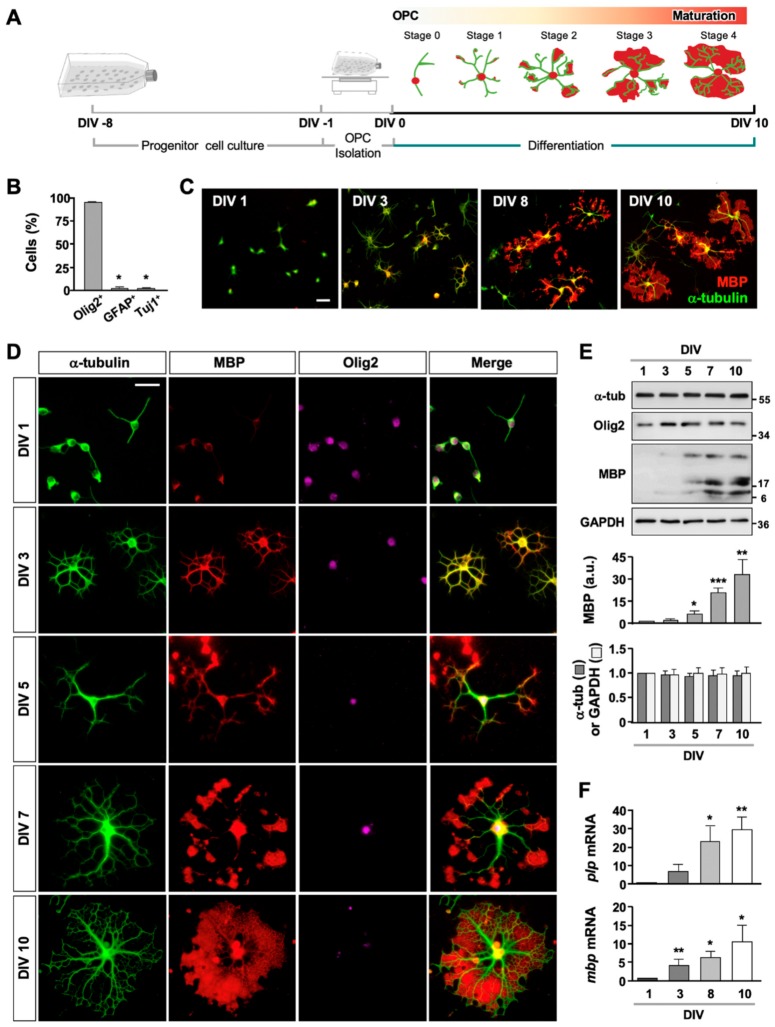
Isolation and differentiation of mouse OPCs. (**A**) Schematics and timeline for isolation and differentiation of oligodendrocytes from mouse OPCs. (**B**) Cell population at DIV 1. Cells were fixed and stained with anti-Olig2, anti-GFAP, and anti-TuJ1 antibodies, and percentage of cells was quantified. Graph shows mean ± SEM. *n* = 4. (**C**) Representative images of oligodendrocyte cultures. Cells were fixed at DIV 1, 3, 8, and 10 and stained for anti-α-tubulin and anti-MBP antibodies. Scale bar, 50 µm. (**D**) Representative images of oligodendrocytes stained with anti-α-tubulin, anti-MBP, and anti-Olig2 antibodies. Scale bar, 25 µm. (**E**) Representative immunoblots of oligodendrocyte lysates probed with antibodies against α-tubulin (α -tub), Olig2, MBP, and GAPDH. Quantification of MBP, α-tubulin, and GAPDH is shown. Graph shows mean ± SEM. *n* = 7. (**F**) mRNA levels of myelin-associated genes, *plp* and *mbp* (normalized to *gapdh*). *n* = 8. * *p* < 0.05, ** *p* < 0.01, and *** *p* < 0.001, Mann–Whitney test.

To obtain OPCs, we first dissociated cortical progenitor cells from postnatal day 1 or 2 (P1 or P2) mice and cultured for 7–9 days (Figure 1A). Then, the mixed glial culture was processed through a series of plating and shaking procedure. When we followed the reported procedure for isolating OPCs from rat tissue [14], we found that few mouse OPCs survived, and the remaining OPCs rarely differentiated (Figure S1). We thus modified the protocol by reducing both the speed and duration of shaking to maximize the survival of mouse OPCs. With the modification, 98.6 ± 0.72% of cells survived, and 95.41 ± 0.29%, 2.35 ± 0.47%, and 2.23 ± 0.37% of the cells were Olig2^+^, GFAP^+^ and TuJ1^+^, respectively (Figure 1B). The purified OPCs were then induced to differentiate by adding differentiating medium (see Materials and Methods for composition). Immunostaining of the cultures with antibodies against myelin basic protein (MBP), which is one of the two major protein components of myelin, showed that both the percentage of MBP^+^ Olig2^+^ double-positive cells and the extent of MBP expression in each Olig2^+^ cell increased with increasing days in vitro (Figure 1C,D). Immunoblotting of the culture lysates also showed the gradual increase in MBP expression (Figure 1E), indicative of differentiation. 

To further assess oligodendrocyte differentiation in culture, we performed quantitative real-time polymerase chain reaction (qRT-PCR) analysis for *proteolipid protein* (*plp*) and *mbp* which encode the two major protein components of CNS myelin. Both mRNAs significantly increased (Figure 1F), confirming that the OPCs purified from mouse brain underwent differentiation in culture. 

### 2.2. Effect of MTAs on Oligodendrocyte Survival and Differentiation

**Figure 2:**
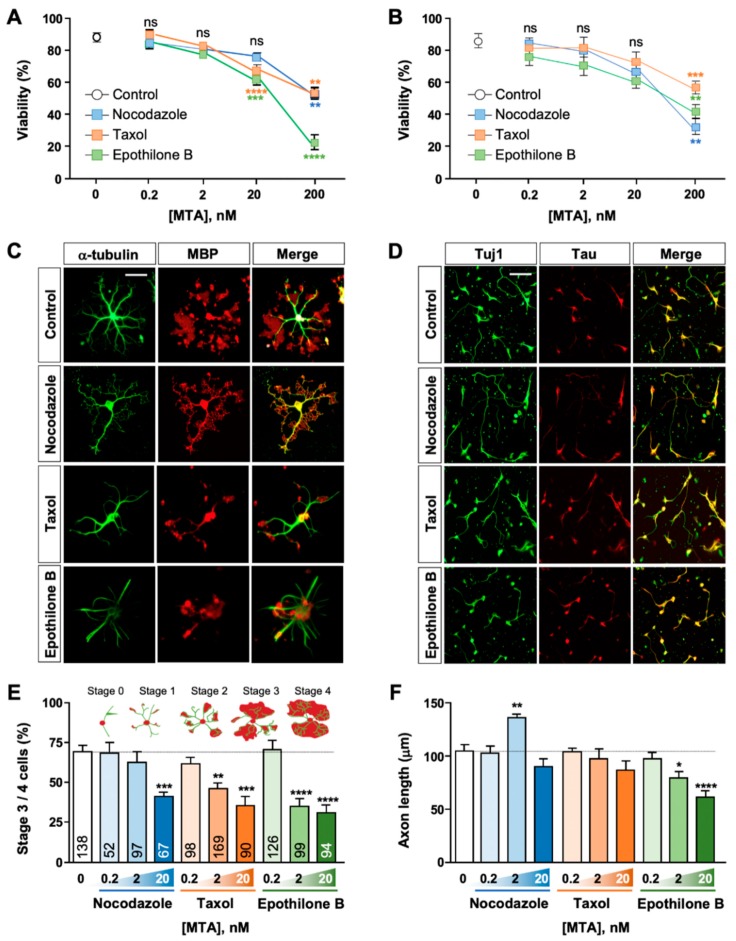
Effects of MTAs in oligodendrocytes and neurons. (**A**,**B**) Cell viability of oligodendrocytes (**A**) or cortical neurons (**B**) cultured in the absence or presence of varying concentrations of MTAs. In oligodendrocytes, MTAs were treated at DIV 3 until fixation at DIV 10. In neurons, MTAs were treated at 5–6 h after plating until fixation at DIV 3. Cell viability was quantified as described in Materials and Methods. Graph shows mean ± SEM. *n* = 12 in (**A**), *n* = 5 in (**B**). (**C**,**D**) Representative images of oligodendrocytes (**C**) and cortical neurons (**D**) treated with MTAs (2 nM) or vehicle control. Scale bar, 25 µm. (**E**) Percentage of differentiated oligodendrocytes (stages 3 and 4) cultured in the absence or presence of varying concentrations of MTAs. Inset, morphological classification of differentiating oligodendrocytes. Detailed criteria for each stage are described in the text. Graph shows mean ± SEM. Total numbers of cells pooled from at least four independent experiments are depicted in each bar. (**F**) Quantification of axon length from cortical neurons cultured in the absence or presence of varying concentrations of MTAs. Graph shows mean ± SEM. *n* = 5. * *p* < 0.05, ** *p* < 0.01, *** *p* < 0.001, and **** *p* < 0.0001; ns, statistically not significant. Student’s *t*-test in (**A**,**B**,**E**), and Mann–Whitney test in (**F**).

We noticed that as oligodendrocytes underwent differentiation, MT structures became highly complex (see Figure 1D). To investigate if MTs played a role in oligodendrocyte differentiation, we treated differentiating oligodendrocytes with varying concentrations of MT-targeting agents (MTAs) (Figure 2). Among specialized cells with complex morphology, neurons have been perhaps the most extensively studied cell type to understand how MTs affect morphogenesis and differentiation [15,16]. MTs in neurons undergo dynamic assembly and disassembly, and the dynamic nature of MTs is essential to neuronal survival and differentiation [17,18].

In both oligodendrocytes and neurons, MTA treatment reduced cell viability in a concentration-dependent manner, all exerting potent cytotoxicity at 200 nM (Figure 2A,B). Notably, epothilone B and taxol reduced oligodendrocyte viability at 20 nM, a concentration which did not significantly affect neuronal cell viability (Figure 2A,B). We then examined the effect of MTAs on oligodendrocyte differentiation (Figure 2C,F). For this purpose, we categorized differentiating oligodendrocytes into 5 stages on the basis of oligodendrocyte morphology and the pattern of MBP expression (see Figure 1A): stage 0 referred to cells extending only one or two primary processes from the cell body and with MBP expression restricted to the soma; stage 1 to cells with multiple processes (three or more) and with MBP expression found in the main branches as well as the cell body; stage 2 to cells with multiple processes and with MBP immunostaining starting to show fan-shaped spreading at the distal ends of the branches; stage 3 to cells showing MBP expression further spread to the extent connecting neighboring main branches; and finally stage 4 to cells with extensive branches and with MBP expression spread to the extent that blurred the boundaries among main branches. Stage 3 and 4 cells were considered differentiated oligodendrocytes. We found that MTAs markedly prevented oligodendrocyte differentiation, and all MTAs tested inhibited differentiation at 20 nM (Figure 2E). At the dose of 2 nM, the concentration which had little effect on cell viability (see Figure 2A), taxol and epothilone B substantially reduced stage 3 and 4 cells (Figure 2E). For comparison, we treated primary cortical neurons with varying concentrations of MTAs side-by-side (Figure 2B,D,F) and examined the effects on axon growth. With the exception of nocodazole (which promoted axon growth at 2 nM), we found that oligodendrocytes exhibited higher sensitivity to all MTAs tested (Figure 2E,F). Micromolar concentrations of MTAs stabilize or destabilize MT and alter MT mass, but nanomolar drug concentrations do not change the overall MT mass but disrupt MT dynamics [19]. Together, these results show that oligodendrocytes are no less vulnerable to disruption of MT dynamics than neurons and suggest that oligodendrocytes require proper regulation of MT dynamics for survival and differentiation.

### 2.3. MT Arborization and MBP Expression

During oligodendrocyte differentiation, MT structures became highly complex, and careful examination revealed that the complex arborization of MTs were tightly associated with the pattern of MBP expression: Most, if not all, MBP immunostaining was detected around the extensive network of MTs (see Figure 1D). This was particularly evident in the fan-shaped MBP spread at the distal ends of stage 2 and 3 cells, when fine structures of MTs started to develop and became elaborated.

**Figure 3:**
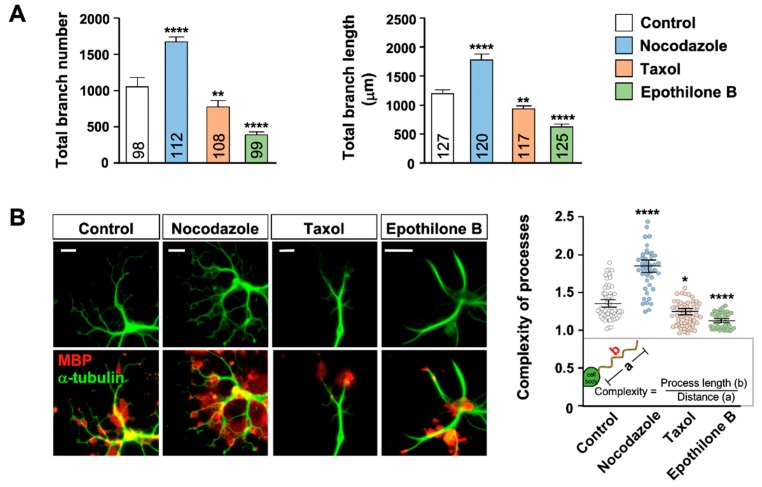
Effect of MTAs on oligodendrocyte morphology. (**A**,**B**) Oligodendrocytes were treated with MTA (2 nM) and immunostained with anti-α-tubulin and anti-MBP antibodies. (**A**) Number of branches of individual oligodendrocytes (**left**) and total branch length (**right**) were quantified. Graphs show mean ± SEM. Total numbers of cells pooled from at least seven independent experiments are depicted in each bar. (**B**) Representative images (**left**) and quantification of process complexity (**right**) of control and MTA-treated oligodendrocytes. Complexity was defined as the actual length of the process divided by the distance from the starting point of main branch to the distal tip. Graph shows mean complexity ± SEM of 42–75 cells pooled from at least four independent experiments. * *p* < 0.05, ** *p* < 0.01, and **** *p* < 0.0001, Student’s *t*-test.

**Figure 4:**
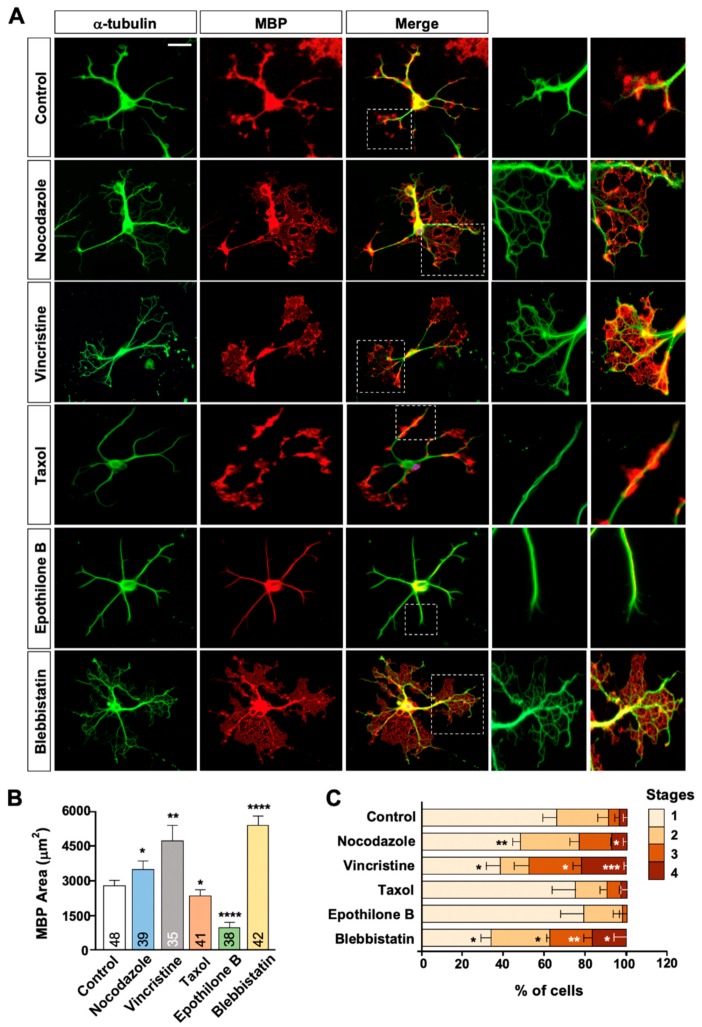
MTAs affect MT arborization, MBP expression, and oligodendrocyte differentiation. (**A**–**C**) Oligodendrocytes were treated with 2 nM of each MTA at DIV 3. Cells were fixed at DIV 5 and immunostained with anti-α-tubulin and anti-MBP antibodies. (**A**) Representative images of control and MTA-treated oligodendrocytes. White boxes are enlarged at right. Scale bar, 25 µm. (**B**) Quantification of area occupied by MBP immunostaining in each oligodendrocyte. Graph shows mean ± SEM. Total number of cells pooled from at least three independent experiments are depicted in each bar. (**C**) Quantification of differentiation stages. Morphological criteria of differentiation stage are identical to Figure 1A. Graph shows mean ± SEM. *n* = 3. * *p* < 0.05, ** *p* < 0.01, *** *p* < 0.001, and **** *p* < 0.0001. Mann–Whitney test in (**B**,**C**).

To further investigate the possible relationship, we treated the cultures with 2 nM of MTAs, a dose which did not affect cell viability. We also reduced the duration of MTA treatment to two days (as opposed to 7 day-treatment in Figure 2) to minimize potential cytotoxicity. We found that total number and length of oligodendrocyte branches (Figure 3A) as well as the complexity of the processes (Figure 3B) were markedly increased by nocodazole but decreased by taxol and epothilone B. Importantly, the area occupied by MBP immunostaining in each cell (Figure 4A,B) as well as the percentage of cells with advanced stages of differentiation (Figure 4C) were markedly increased in cells treated with nocodazole for two days, as compared to control. Similarly, vincristine promoted MT arborization and MBP distribution in each cell (Figure 4A,B) and enhanced oligodendrocyte differentiation (Figure 4C). By contrast, epothilone, which greatly simplified MT arborization, drastically reduced the area occupied by MBP immunostaining (Figure 4A,B) and inhibited oligodendrocyte differentiation (Figure 4C), as compared to control. A similar trend was induced by two-day-taxol treatment, but the effects on differentiation did not reach to statistically significant values. Blebbistatin is a selective inhibitor of non-muscle myosin II [20,21] and can promote oligodendrocyte differentiation [22]. Blebbistatin also substantially increased MBP area in each cell, which was again tightly associated with the extensive elaboration of MT network (Figure 4A,B). Together, these findings suggest that formation of MT network regulates the expression or distribution pattern of MBP, and hence oligodendrocyte differentiation.

### 2.4. A Role of MT Dynamics in the Maintenance of Myelination

**Figure 5:**
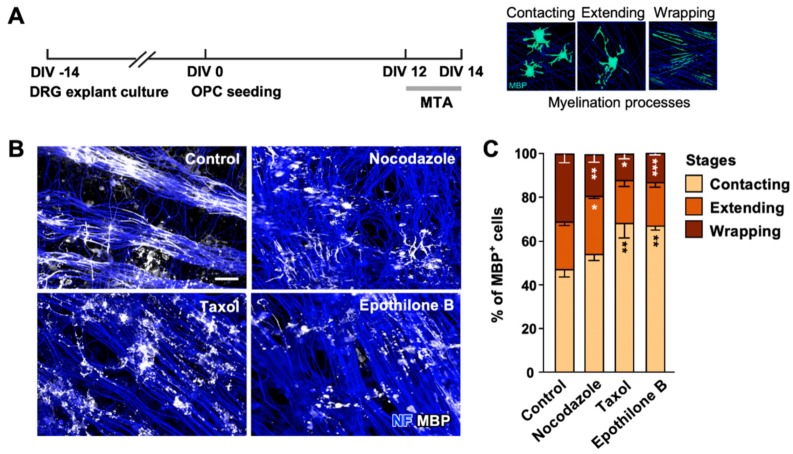
MT dynamics is essential for myelination. (**A**) Experimental timeline of myelinating co-cultures of OPCs and dorsal root ganglion (DRG) explants (**left**). Illustrations of myelinating oligodendrocytes are shown on **right**. DRGs were cultured and maintained for 14 days, and purified OPCs were added to the DRG explant culture at DIV 0. Co-cultures were treated with MTAs (2 nM) at DIV 12 and fixed at DIV 14. Myelination proceeds through three stages, contacting, extending, and wrapping. (**B**) Representative images of co-cultures stained with anti-neurofilament (NF) and anti-MBP antibodies. Scale bar, 25 µm. (**C**) Quantification of myelination. Myelinating oligodendrocytes were categorized as depicted in A. Graph shows mean ± SEM. *n* = 4. * *p* < 0.05, ** *p* < 0.01, and *** *p* < 0.001, Mann–Whitney test.

Our results so far suggest that development of the complex MT network plays an essential role in oligodendrocyte differentiation. To examine if MTs continued to play a role in myelination, we co-cultured oligodendrocytes with explants of dorsal root ganglion (DRG) (Figure 5A). In control, differentiating oligodendrocytes co-cultured with the DRG explants, first contacted axons, extended their processes, and eventually wrapped axonal segments within 2 weeks (Figure 5A). We applied 2 nM of MTAs at DIV 12 when 47.18% ± 2.93%, 21.30% ± 1.47%, and 31.52% ± 2.13% cells were underdoing stages of contacting, extending, and wrapping, respectively. All MTAs tested substantially reduced the percentage of cells undergoing wrapping (Figure 5B,C), suggesting that MT dynamics continue to play a role at later stages of oligodendrocyte differentiation and myelination.

## 3. Discussion

In oligodendrocytes, F-actin disassembly has been suggested to drive the expansion of myelin sheets [5,6,23], and myelinating oligodendrocytes seem to lack the intermediate filament system [9,24], which leaves only the MT cytoskeleton, especially at later stages of differentiation. In this study, we have shown that (i) oligodendrocytes develop a complex network of MTs during differentiation and suggest that (ii) altering MT dynamics in differentiating oligodendrocytes by treating cells with nanomolar concentrations of MTAs has a profound impact on cell viability, differentiation, and myelination. All MTAs tested reduced cell viability and prevented differentiation when treated for a week (see Figure 2), but acute exposure to nanomolar concentrations of MTAs for a couple of days produced opposing outcomes depending on the type of MTAs (see Figure 3 and Figure 4). We found that nocodazole and vincristine treatment promoted oligodendrocyte differentiation, whereas taxol and epothilone inhibited differentiation. Although it is an overly simplistic classification, MTAs can be divided into stabilizers (taxanes and epothilones) and destabilizers (vinca alkaloids and nocodazole) [25], based on their effects on MT mass at high concentrations. At micromolar (high) concentrations, stabilizers stimulate MT polymerization, whereas destabilizers inhibit it. At nanomolar concentrations, however, both the stabilizers and the destabilizers alter MT dynamics without changing the mass of MTs. Vincas and nocodazole, at low concentrations, are known to kinetically stabilize MTs, but MTs stabilized in this fashion can still undergo some level of subunit exchange, allowing both the assembly and the disassembly of tubulin subunits [26]. Therefore, those MTs are not stabilized in the same way as taxol-stabilized MTs, which might provide an explanation for the opposing effects on oligodendrocyte differentiation. The mechanism by which nocodazole and vincristine enhanced differentiation is not clear at this point, but it seems that continued exchange of tubulin subunits is required for both the survival and the differentiation of oligodendrocytes.

In recent years, MTs have emerged as a promising target to treat nerve injury and neurodegenerative diseases [27], and applying MTAs in animal models of spinal cord injury, Alzheimer’s disease, and Parkinson’s disease has yielded promising results [28,29,30,31]. In fact, MTAs have been used for decades in the treatment of cancer, and MTAs continue to be among the most effective anticancer drugs. Unfortunately, MTAs are well known to cause significant side effects, including long-term neurological deficits such as cognitive dysfunction and impaired attention, fine motor and executive function. Given that neurons are vulnerable to MTAs [32,33], chemotherapy-related neurological deficits, colloquially known as “chemobrain” are often regarded as consequences of neuronal dysfunction or degeneration of neurons. In this study, by performing experiments in oligodendrocytes and neurons side-by-side, we found that oligodendrocytes were no less vulnerable to MTA exposure as compared to neurons, in terms of both survival and differentiation. Furthermore, in the neuron-oligodendrocyte co-culture, low dose of MTA treatment severely affected oligodendrocytes and induced dysmyelination or demyelination, while leaving the underlying axons relatively intact. Indeed, increasing lines of evidence suggest that chemotherapy-related cognitive impairment is related to white matter damages, implying dysregulation of oligodendrocyte-lineage cells [34,35,36]. Now, there is considerable attention to repurposing (or repositioning) of MTAs for the treatment of a variety of brain disorders, with some already in clinical trials [27,37]. Results from this paper support the notion that in addition to neurons, the effects of MTAs on oligodendrocytes should be thoroughly investigated before applying MTAs to brain disorders in order to prevent adverse effects and exploit the full benefit of drug repurposing.

We found that MBP expression was tightly associated with MT arborization in oligodendrocytes: MBP immunostaining was detected around the fine arborization of MTs, and altering MT dynamics with MTAs induced changes in the expression pattern of MBP in a manner that correlated with how the drugs modified MT arborization. MBP is a major protein of CNS myelin, comprising 30%-40% of the total protein and ~10% of the dry weight of myelin [38,39]. The importance of MBP is underscored by the severe dysmyelination and shortened lifespan of *shiverer* mice which contain a deletion in the *Mbp* gene [40]. MBP plays an essential role in the organization of myelin sheath (i) by zippering up adjacent layers of the myelin, which contributes to the compaction of myelin [38], and (ii) by functioning as a molecular sieve that restricts the entry of proteins into myelin sheath to generate a lipid-rich plasma membrane [12]. While substantial progress has been made in understanding the role of MBP in organizing the components and structure of myelin, little is known about the mechanisms of how the expression or organization of MBP itself is regulated. Given that diffusion of protein is limited in myelinating oligodendrocytes [12] and that MBP is sufficient to compact cell membranes both in vivo and in vitro [41], it is important to deliver *Mbp* mRNA to proper regions of the cell, i.e., distal processes where myelination occurs. *Mbp* mRNA has been suggested to be transported into the processes of myelinating oligodendrocytes via a MT-based motor protein KIF1B, and *Kif1b* mutation in zebrafish causes accumulation of MBP protein in the cell body and abnormal myelination [42]. Moreover, *KIF1B* has been associated with susceptibility of multiple sclerosis [43]. A recent study has suggested that TPPP nucleates MTs in oligodendrocytes and that TPPP is essential for the elongation of myelin sheaths [11]. Although future studies are needed to understand exactly how MTs play a role in myelination, these lines of evidence and our results suggest that MT organization and perhaps the MT-based trafficking system plays a critical role in oligodendrocyte differentiation, in part, by controlling the distribution or expression pattern of MBP. Further knowledge on the mechanism by which MT network is intertwined with the expression and localization of myelin proteins might provide insight into the cell biological mechanisms of myelination.

## 4. Materials and Methods

### 4.1. Animals

ICR mice were used for primary cultures of mixed glial cells, cortical neurons and embryonic dorsal root ganglia (DRG) explant cultures. Mice were purchased from DBL (Eumseong, South Korea), and housed, bred, and treated with the approval and in accordance with the research protocols approved by the Institutional Animal Care and Use Committee of Seoul National University and Korea Institute of Science and Technology (Approval code: KIST 2017-065, SNU-180518-3, SNU-190413-4: 29.11.2016, 19.7.2018, 5.6.2019).

### 4.2. Mixed Glial Cell Culture

Primary mixed glial cells were isolated from cerebral hemispheres of P1 to P2 mice. Briefly, dissected brains were freed of meninges and treated with 0.05% trypsin (Sigma-Aldrich, T1426, St. Louis, MO, USA) in culture media (Dulbecco′s modified Eagle medium (DMEM) (Hyclone, sh30243.01) containing 10% (*w*/*v*) fetal bovine serum (FBS) (Thermo Fisher Scientific, 16000-044, Waltham, Massachusetts, United States) and 1% penicillin-streptomycin (Gibco, 15140-122)) for 15 min at 37 °C. The tissue was then incubated in DMEM supplemented with 20% (*w*/*v*) FBS and 10 U/µL DNase I (Sigma-Aldrich, D5025) for 10 min at RT, followed by gentle trituration. The dissociated tissue was filtered through a Falcon 100 μm cell strainer (Corning Inc., 352360, Corning, New York, United States) and then centrifuged for 10 min at 1000 rpm. The pellet was resuspended in culture media, filtered through a Falcon 70 µm cell strainer (Corning Inc., 352350), and centrifuged for 10 min at 800 rpm. The pellet was resuspended with culture media, filtered through a Falcon 40 μm cell strainer (Corning Inc., 352340), and plated on 75 cm^2^ flasks (Corning Inc., 430720U) coated with 10 g/mL poly-d-lysine (Sigma-Aldrich, P6407) at a density of 1 × 10^7^ cells/75 cm^2^. Culture media was changed every 2–3 days for 7–9 days and cultured in a 5% CO_2_ humidified incubator at 37 °C.

### 4.3. Primary Culture of OPCs from Mouse Brain

OPCs were isolated from mixed glial cells. Briefly, mixed glial cells cultured for 7–9 days were pre-shaken at 200 rpm for 1 h at 37 °C. Medium was replaced, and the cells attached were incubated for 4–5 h at 37 °C. The cells were then processed through overnight shaking at 260 rpm at 37 °C. Cell suspension was collected and centrifuged (1500 rpm, 10 min, RT). The pellet was treated with 0.25% trypsin-EDTA (Gibco, 25200056) for 5 min at RT. Cell suspension was then incubated in DMEM containing 20% (*w*/*v*) FBS for 10 min at RT. The pellet was collected by centrifugation (1500 rpm, 10 min, RT), resuspended in culture media, and then plated on a petri dish (SPL, 10090), followed by incubation for 1 h at 37 °C. Final cell suspension obtained after three rounds of differential adhesion steps was passed through a 40 m cell strainer and plated on a 35-mm culture dish or a 24-well plate containing 12-mm glass coverslips (Marienfeld, 0111520, Lauda-Königshofen, Germany) coated with 10 g/mL poly-d-lysine, at a density of 5.5 × 10^6^/dish or 8 × 10^4^/well. On the following day, media was completely replaced with the differentiation medium (DMEM containing 50 g/mL transferrin (Millipore, 82-057, Burlington, Massachusetts, United States), 20 g/mL putrescine (Sigma-Aldrich, P5780), 12.8 ng/mL progesterone (Sigma-Aldrich, P8783), 10.4 ng/mL sodium selenite (Sigma-Aldrich, S5261), 25 g/mL insulin (Sigma-Aldrich, I5500), 0.8 g/mL 3,3′,5,5′-tetraiodo-l-tyronine (Sigma-Aldrich, T1775), 6.6 mM L-glutamine (Gibco, 25030) and 6 g/mL D-glucose (Sigma-Aldrich, G7021)). One-half of differentiation medium was changed every 2–3 days.

### 4.4. Cortical Neuron Culture

Primary cortical neurons were cultured following a previous protocol [32]. Briefly, cortices from P1 mouse brain were dissected and incubated in papain solution (20 U/mL, Worthington, LS003120, Lakewood, New Jersey, United States) containing 10 U/mL DNase I for 30 min at 37 °C. The cortices were washed three times with HBSS, and then triturated with HBSS supplemented with 10% (*w*/*v*) FBS. Dissociated neurons were collected by centrifugation (1000 rpm, 5 min, RT). Cell pellet was resuspended in neuronal plating media (Neurobasal medium (Corning Inc, 21103-049) containing 2% (*w*/*v*) B-27 supplement (Thermo Fisher Scientific, 17504044) and 2 mM l-glutamine) and plated on a 24-well plate containing coverslips coated with 10 g/mL poly-d-lysine at a density of 8.5 × 10^4^/well and then incubated at 37 °C in a 5% CO_2_ humidified incubator. Neurons were treated with indicated drugs at 5–6 after plating.

### 4.5. Myelinating Co-culture of Mouse OPCs and Dorsal Root Ganglion (DRG) Neurons

For myelinating neuron-oligodendrocyte co-cultures, OPCs were seeded on DRG explant cultures. DRGs were purified from embryonic day 13.5 ICR mouse and cultured as previously described [21], with minor modifications. Briefly, thoracic and lumbar DRGs were plated on coverslip coated with matrigel (Corning Inc, 356230) diluted with Neurobasal media. Explant cultures were maintained in DRG plating medium (Neurobasal medium containing 2% (*w*/*v*) B-27 supplement, 2 mM l-glutamine, 50 ng/mL nerve growth factor (Alomone labs, N-100, Jerusalem, Israel), and 1% penicillin-streptomycin) for 3 days. Then medium was replaced with DRG maintaining medium (Neurobasal medium containing 2% (*w*/*v*) B-27 supplement, 50 ng/mL nerve growth factor, and 1% penicillin-streptomycin) for 2 weeks. OPCs were seeded on DRG explant cultures at a density of 1 × 10^5^/well in a 24-well plate. On the following day, medium was completely switched to co-culture medium (1:1 of differentiation medium and DRG maintaining media) and changed every 3 days until fixation.

### 4.6. Drug Treatment

Paclitaxel (Cell Signaling Technology, 9807S, Danvers, Massachusetts, United States), epothilone B (Abcam, ab141271, Cambridge, United Kingdom), and nocodazole (Abcam, ab120630) stock solutions were prepared in dimethyl sulfoxide (DMSO) and diluted with differentiation media, neuron plating media, or co-culture media, depending on the culture. Drugs of the indicated concentrations were added to the culture by exchanging one-half of media.

### 4.7. Immunofluorescence

Immunostaining was performed as described elsewhere [32,44]. Cells were fixed with pre-warmed 4% (*w*/*v*) paraformaldehyde (PFA), 0.15% glutaraldehyde, and 0.1% (*w*/*v*) Triton X-100 dissolved in phosphate buffered saline (PBS) for 15 min at 37°C. After washing with PBS twice, cells were treated with 0.1% (*w*/*v*) sodium borohydride (Honeywell Fluka, 71321, Charlotte, North Carolina, United States) dissolved in PBS for 20 min at RT. Primary antibodies were diluted in blocking solution (3% (*w*/*v*) bovine serum albumin (BSA) and 0.1% (*w*/*v*) Triton X-100 in PBS). The following primary antibodies were used: anti-Olig2 antibodies (Merck Millipore, AB9610), anti-MBP antibodies (Abcam, ab7349), anti-tubulin antibodies (Sigma-Aldrich, T6199). Alexa fluor conjugates (Thermo Fisher Scientific, A11007, A28175 and A21244) were used as secondary antibodies. Nuclei were stained with Hoechst (Molecular Probes, 33342). All images were acquired with an inverted light microscope (Axio Observer Z1, Carl Zeiss MicroImaging, Inc., Jena, Germany) equipped with epifluorescence optics. Images were captured with a CCD camera controlled by ZEN software (Carl Zeiss MicroImaging, Inc.). Areas stained for MBP and axon length were drawn or traced manually by using draw spline contour and curve graphic elements of the ZEN software (Carl Zeiss Micro Imaging, Inc.).

### 4.8. Immunoblotting

Lysates from cultured cells were collected in RIPA buffer (Sigma-Aldrich, R0278) containing phosphatase and protease inhibitors (Sigma-Aldrich, P8340, P5726, and P0044). Protein lysates were mechanically homogenized and centrifugated at 1200 rpm for 5 min at 4 °C, and protein concentrations were determined by Pierce™ BCA assay (Thermo Fisher Scientific, 23228). Lysates were loaded onto SDS-PAGE and transferred on PVDF membrane (Bio-Rad, 1620177). Membranes were blocked with 5% skim milk in TBS-T (25 mM Tris-HCl, 150 mM NaCl and 0.1% (*w*/*v*) Tween-20, pH 7.4) at RT and probed with primary and secondary antibodies. Bands were detected by using the Clarity Western ECL substrate (Bio-Rad, 1705060, Hercules, California, United States) and developed by using chemiluminescent imaging system (Vilber Lourmat, Solo 6S, Eberhardzell, Germany), according to the manufacturer’s instructions. Intensity of protein bands were quantified by using Image J software and the results were normalized against the density of -tubulin bands.

### 4.9. RNA Preparation and Quantitative Real-time Polymerase Chain Reaction (qRT-PCR)

Total RNA was extracted by using Trizol (Life technologies, 15596018, Carlsbad, California, United States), according to the supplier’s instructions and reverse-transcribed into cDNA (Promega, ImProm-II Reverse transcription system, A3800, Madison, Wisconsin, United States). Transcripts were amplified by qRT-PCR using SYBR Green Master mix (Enzynomics, RT501M) in Quantstudio 3 (Thermo Fisher scientific), according to the manufacturer’s instructions. The mRNA expression data were normalized against the level of *glyceraldehyde 3-phosphate dehydrogenase* (*gapdh*) mRNA. The sequences of forward and reverse primers were as follows: *mbp*, F 5′-atccaagtacctggccacag-3′ and R 5′-cgaagaaatcgccactgtcc-3′; *plp*, F 5′-acctggaccacctgtcagtc-3′ and R 5′-aagagggtaccttacgaaag-3′; and *gapdh*, F 5′-aactttggcattgtggaagg-3′ and R 5′-tcttgtagtagggacgtagg-3′.

### 4.10. Cell Viability Assay

Cell viability was determined by incubating cells in differentiation media or neuron plating media containing 10 g/mL propidium iodide (PI, Thermo Fisher scientific) for 20 min at 37 °C in a 5% CO_2_ humidified incubator. The cells were fixed and the number of PI-positive cells were quantified. Cells were stained with anti-tubulin antibodies (III for neurons and -tubulin for oligodendrocytes) to visualize cell morphology. At least 5 random fields were quantified from each culture well. Total cell number was counted with a Hoechst dye or by immunostaining.

### 4.11. Data Analysis and Statistic

All statistical analysis was performed using GraphPad Prism 8 Software (GraphPad Software, Inc., San Diego, California, United States). Before determining statistical significance, a Shapiro–Wilk test was performed to assess normality. Student’s *t*-test was used to compare the means between two samples (Figure 2A,B,E, Figure 3A,B and Figure 4B). For non-normal variables, the Mann–Whitney test was used to determine statistical significance (Figure 1E,F, Figure 2F,C and Figure 5C). All statistical analyses were conducted with data from a minimum of three independent experiments, and data were presented as mean ± standard error of the mean (SEM). The level of statistical significance was set at *p* < 0.05.

## Supplementary Materials

Supplementary Figure S1 can be found at https://www.mdpi.com/1422-0067/21/3/1062/s1.

## Abbreviations

CNS	Central nervous system
GAPDH	Glyceraldehyde 3-phosphate dehydrogenase
DIV	Day in vitro
DRG	Dorsal root ganglion
MBP	Myelin basic protein
MT	Microtubule
MTA	Microtubule-targeting agent
NF	Neurofilament
OPC	Oligodendrocyte precursor cell
PLP	Proteolipid protein
TPPP	Tubulin polymerization promoting protein
