# Supplementary material for: A Role of Microtubules in Oligodendrocyte Differentiation
Source: Int J Mol Sci. 2020 Feb 5;21(3):1062. doi: 10.3390/ijms21031062 (PMC7037135; doi:10.3390/ijms21031062)
Supplement: Supplementary file 1 [file ijms-21-01062-s001.pdf]

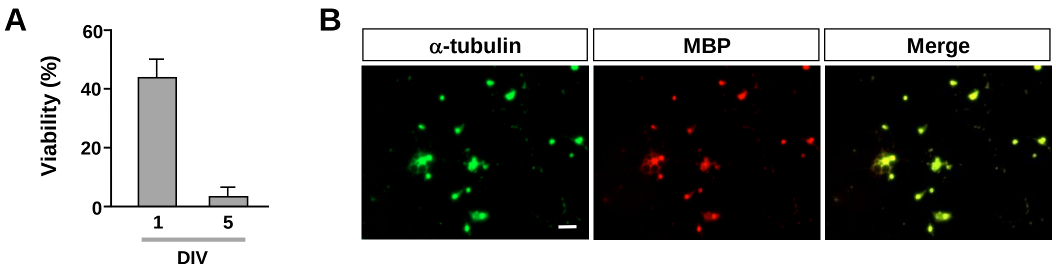

Figure S1. Viability and differentiation of mouse OPCs following a protocol for isolating and differentiating rat OPCs. **(A)** Cell viability of differentiating oligodendrocytes at DIV 1 and 5. Graph shows mean ± SEM. N = 5. **(B)** Representative images of oligodendrocytes at DIV 5 stained with anti-α-tubulin and anti-MBP antibodies. Scale bar, 25 μm.
